# Carcinogenic Risk of Pb, Cd, Ni, and Cr and Critical Ecological Risk of Cd and Cu in Soil and Groundwater around the Municipal Solid Waste Open Dump in Central Thailand

**DOI:** 10.1155/2022/3062215

**Published:** 2022-02-28

**Authors:** Paweena Aendo, Ramnaree Netvichian, Piriyaporn Thiendedsakul, Sutha Khaodhiar, Phitsanu Tulayakul

**Affiliations:** ^1^Faculty of Veterinary Medicine, Kasetsart University, Bangkok 10900, Thailand; ^2^Department of Environmental Engineering, Faculty of Engineering, Chulalongkorn University, Bangkok 10330, Thailand; ^3^Animal Health and Biomedical Science, Faculty of Veterinary Medicine, Kasetsart University, Bangkok 10900, Thailand; ^4^Center of Excellence on Hazardous Substance Management, Chulalongkorn University, Bangkok 10330, Thailand; ^5^Department of Veterinary Public Health, Faculty of Veterinary Medicine, Kasetsart University, Kamphaeng Saen Campus, Nakhon Pathom 73140, Thailand

## Abstract

Several consequences of health effects from municipal solid waste caused by carcinogenic and noncarcinogenic metals have been recognized. The water quality index (WQI) in the groundwater around this landfill is 2945.58, which is unacceptable for consumption. The contaminated groundwater mainly appears within a 1 km radius around the landfill. The metal pollution levels in the soil in descending order were Cu > Cd > Zn=Cr > Pb > Ni. The pollution degree (ER) of Cd was 2898.88, and the potential ecological risk index (RI) was 2945.58, indicating that the risk level was very high. Surprisingly, the hazard index (HI) of Pb (2.05) and Fe (1.59) in children was higher than 1. This indicated that the chronic risk and cancer risk caused by Pb and Fe for children were at a medium level. Carcinogenic risk by oral (CR oral) consumption of Ni, Cd, and Cr in children was 1.4*E* − 04, 2.5*E* − 04, and 1.8*E* − 04, respectively, while the lifetime carcinogenic risk (LCR) of Ni, Cd, and Cr in children was 1.5*E* − 04, 2.8*E* − 04, and 2.0*E* − 04, respectively. In adults, CR oral of Ni and Cr were 1.6*E* − 03 and 3.0*E* − 04, respectively, while LCR of Ni and Cr were 1.6*E* − 03 and 3.4*E* − 04, respectively, which exceeded the carcinogenic risks limits. Our study indicated a lifetime carcinogenic risk to humans. Environmental surveillance should focus on reducing health risks such as continuous monitoring of the groundwater, soil, and leachate treatment process.

## 1. Introduction

The municipal solid waste open dump is considered to constitute a major problem for groundwater, surface water, and soil contamination caused by potentially hazardous chemicals such as heavy metals [1]. A significant portion of a landfill's leachate incorporates heavy metals (Mercury (Hg), Arsenic (As), Zinc (Zn), Lead (Pb), Nickel (Ni), Cadmium (Cd), Manganese (Mn), Iron (Fe), Cobalt (Co), Chromium (Cr), Aluminum (Al), and Copper (Cu)), which is readily soluble and constant in concentration during degradation processes [2, 3]. Also, heavy metals are a critical threat to soil quality due to their permanence in the soil [4]. Furthermore, heavy metals in soil can be easily transferred into the human body via the oral route (ingestion), dermal contact absorption, and inhalation [5]. Exposure to heavy metal contamination soils through hand-to-mouth contact causes adverse human health effects, particularly in children [6]. Since the 1970s, groundwater contaminated by landfill waste has been a concern in many countries, especially developing countries which are attributed to poor transportation, lack of waste management practices, uncontrolled dumping of municipal solid waste, and disposal of municipal solid waste open dump, which are unscientific and unplanned [7, 8].

Currently, the concentrations of heavy metals in soil have been determined in several parts of the world. The major indices revealing the degree of heavy metal pollution in the soil, sediments, and water were the geoaccumulation, ecological risk factor, potential ecological risk index, and water quality index. Conversely, the potential ecological risk index in the soil comprehensively considers the concentration of biotoxicity and migration regularity, associates ecological toxicology, and evaluates the pollution [9]. Health risk assessment is assigned as a process of estimating the possibility of an occurrence of any adverse health effects in humans throughout the hazard chemical exposure and is usually based on an expression in parts of noncarcinogenic, carcinogenic health risk, and measurement of the risk level [10]. Exposure to carcinogenic or noncarcinogenic metals can result in adverse health consequences including damage to the nervous system, bone fractures, pulmonary adenocarcinomas, cardiovascular disease, kidney and liver dysfunction, and immune systems disorder [11, 12]. According to the classification orders defined by the International Agency for Research on Cancer (IARC), Ni (group 1), Pb (group 2B), Cd (group 1), and Cr (group 1) are appointed as potential carcinogenicity metals. Meanwhile, Zn, Mn, Al, and Cu are appointed as noncarcinogenic metals [13, 14].

In 2018, the amount of waste generated in Thailand was approximately 27.8 million tons. Compared to 2017 volume, this was an increase of 1.64 percent, but 9.58 million tons (34%) were recycled. Hazardous waste content was 638,000 tons, an increasing rate of 3.2% from 2017, which was due to the urbanization of lifestyle, increasing population, and tourism. Thailand has 3,205 community waste disposal sites in various locations, of which 2,785 locations are opening sites and 419 are closed sites [15].

The open dumpsite was not well systematically designed before being used as a dump for municipal solid waste. Furthermore, an environmental impact assessment has not yet been carried out in this study area. Unfortunately, groundwater was mainly used for agriculture and livestock purposes without any testing or treatment. Thus, the aim of this study was to focus on the environmental risks and human health risks posed by this open dump. Therefore, the first aim of this study was to determine the heavy metal contamination, including Zn, Pb, Ni, Cd, Mn, Fe, Cr, Al, and Cu in groundwater, and soil around the municipal solid waste open dump of Lopburi Province. The secondary aim was to assess the water quality index in the groundwater, soil pollution, and the potential ecological risks of heavy metal contaminants in the soil, as well as estimate the carcinogenic risks from heavy metal contaminants in the soil via oral ingestion, dermal exposure, and inhalation.

## 2. Materials and Methods

### 2.1. Sample Collection

**Figure 1:**
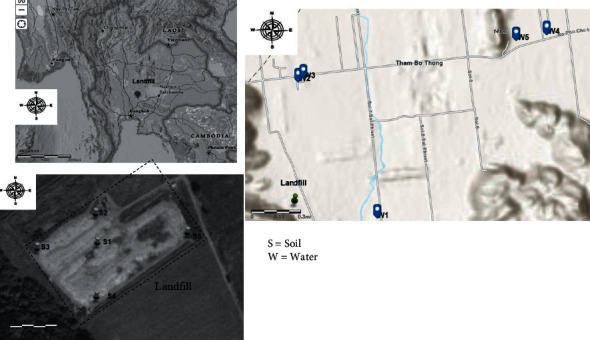
Location of the soil and groundwater samples collected in a municipal solid waste open dump at Lopburi Province, Thailand.

A more than 15-year-old municipal solid waste open dump in Lopburi Province located in the central region of Thailand was selected for this study. The population in this area was about 8,054 people, who generated an estimated 10 tons of garbage daily. The total waste collection per day delivered to the open dump was approximately 16 tons. The study area was located between 14.80348°∼14.80753 north latitude and 100.86082°∼100.88307° east longitude. A one-liter groundwater sample was taken from household tanks (depth∼40–80 m) in five different locations in the range of households residing nearest the landfill, all of which were located <2 km around the municipal solid waste open dump. Groundwater samples were preserved by using 2-3 ml of Conc. HNO_3_ for avoiding the metal precipitation and put in a refrigerator at below 4°C until analysis [16]. One kilogram of pooled topsoil (0–20 cm depth) from five different positions across the landfill was collected, put in fresh polyethylene bags, kept in a refrigerator at below 4°C, and then immediately brought to the laboratory [17]. Five groundwater samples and five soil samples were collected every month from January to December 2017, therefore, a total of 60 samples for groundwater and soil samples were collected for analysis. The sampling locations are depicted in Figure 1.

### 2.2. Analytical Methods

The groundwater samples were digested by using HNO_3_ heated on a block at 150 ± 20°C [18]. The soil samples were air-dried overnight at 105°C and crushed and sieved over a diameter sieve of 2 mm [19–21]. After passing through the sieve, the sample fraction was taken for analysis. The soil was digested by using HNO_3_ and HCl [18]. Subsequently, all the samples were heavy metal measured by using an inductively coupled plasma atomic emission spectrophotometer (ICP-AES) ULTIMA2, Jobin Yvon Horiba, Italy. The limits of detection (LOD) were calculated as the standard deviation of 10 blank solution samples divided by the slope of the calibration curve(mg/L) and were Zn (0.0024), Pb (0.0030), Ni (0.0007), Cd (0.0003), Mn (0.0003), Fe (0.0087), Cr (0.0002), Al (0.0014), and Cu (0.0010), respectively. The relative standard deviation (RSD) was ≤5%. The concentrations of recovered metals were 5, 10, 20, and 50 mg/kg in all samples with 4 replications for each spike level; the recovery rates for the metals were as follows: Zn, 98%; Pb, 93.2%; Ni, 106.8%; Cd, 101%; Mn, 100.29%; Fe, 87.74%; Cr, 93.13%; Al, 106.57%; and Cu, 98.5%.

### 2.3. Determination of the Groundwater Quality Index

Principle component analysis (PCA) was conducted to analyze the information content of the indicators of commonalities of the metal parameters between the variables in the groundwater quality by using NCSS, PASS, and GESS, 2007 (NCSS Statistical Software). The components with eigenvalues <1 have less variation than an individual variable [22].

Then, the quality rating scale (Q*i*) for each parameter was calculated as

(1) $$\begin{array}{c} Q_{i}=\left[\frac{C_{i}}{S_{i}}\right]100, \end{array}$$

where *Q* is the quality rating, *C* is the concentration of each metal in the groundwater sample (mg/L), *S* is the WHO standard for each metal in drinking water [23, 24] as listed in Table 1, and *i* is the quality parameter [33].

The subindex of the *i* parameter was calculated as

(2) $$\begin{array}{c} {\text{SI}}_{i}=W_{i}Q_{i}, \end{array}$$

where SI is the subindex of the *i* parameter, *W* is the relative weight, *Q* = the quality rating, and *i* is the quality parameter.

Finally, the water quality index was calculated as

(3) $$\begin{array}{c} \text{WQI}=\left[\frac{\sum_{i=1}^{n}{\text{SI}}_{i}}{\sum W_{i}}\right], \end{array}$$

where WQI (water quality index) was calculated by using the Weighted Arithmetic Index method [34], and SI is the subindex and the weight of the *i* parameter. The water quality indices are categorized into 5 categories [33].

### 2.4. Pollution Indices

The geoaccumulation index (*I*_*geo*_) was introduced by Mϋller [35], and it is used to quantify and define heavy metal pollution in soil and sediment. The *I*_*geo*_ was computed by using the following equation:

(4) $$\begin{array}{c} I_{geo}=\;\log_{2}\left[\frac{C_{n}}{1.5B_{n}}\right], \end{array}$$

where *C*_*n*_ is the concentration of metal *n* found in the sampled soil (mg/kg), *B*_*n*_ is the geochemical background value (average scale) of metal *n* (mg/kg) as listed in Table 1, and factor 1.5 is the background matrix correlation factor due to lithogenic variation [36]. Mϋller [35] suggested seven classes of the geoaccumulation index.

### 2.5. Indices of the Ecological Risk Assessment

#### 2.5.1. Pollution Coefficient

The pollution coefficient of each metal was derived by employing the model defined by Hakanson and Rahman et al. [28, 37] using the following equation:

(5) $$\begin{array}{c} C_{f}^{i}=\frac{C_{D}^{i}}{C_{R}^{i}}, \end{array}$$

where *C*_*f*_^*i*^ is the pollution coefficient of each metal in the soil or sediment, and *C*_*D*_^*i*^ is the *i* heavy metal concentration (mg/kg) in the soils. *C*_*R*_^*i*^ is the background values (mg/kg) of *i* heavy metal in the soils set as the highest background value of metals in sediments in modern preindustrial times, as suggested by Hakanson [28] and shown in Table 1.

#### 2.5.2. Ecological Risk Factor

Ecological risk factor (*E*_*R*_^*i*^) quantitatively expresses the potential ecological risk of a given single contaminant [28, 38] which can be calculated for each metal by using the following equation:

(6) $$\begin{array}{c} E_{R}^{i}=T_{R}^{i}\times C_{f}^{i}, \end{array}$$

where *C*_*f*_^*i*^ is the pollution coefficient for each metal and *T*_*R*_^*i*^ is the toxic response coefficients of the metals in the water, sedimentary, and biological phases, as listed in Table 1.

#### 2.5.3. The Potential Ecological Risk Index (RI)

The potential ecological risk index (RI) was defined as the sum of the risk factors by using the following equation:

(7) $$\begin{array}{c} \text{RI}=\sum_{i=1}^{m}E_{R}^{i}, \end{array}$$

where RI is the sum of *E*_*R*_^*i*^ for all metals examined, representing the contamination degree of the environment (Hakanson [28]).

### 2.6. Human Health Risk Assessment

#### 2.6.1. Exposure Assessment

The average daily intake (ADI) of heavy metal in soils was expressed as units of the contaminated body exposed per unit of body mass and day [39], calculated using the following equations:

(8) $$\begin{array}{c} {\text{ADI}}_{\text{oral}}=\frac{C\times {\text{IR}}_{\text{ing}}\times \text{EF}\times \text{ED}}{\text{Bw}\times \text{AT}}\times 10^{-6},\\ {\text{ADI}}_{\text{dermal}}=\frac{C\times \text{SA}\times \text{SAF}\times \text{ABS}\times \text{EF}\times \text{ED}}{\text{BW}\times \text{AT}}\times_{10}^{-6},\\ {\text{ADI}}_{\text{inh}}=\frac{C\times \text{IRinh}\times \text{EF}\times \text{ED}}{\text{PEF}\times \text{BW}\times \text{AT}}, \end{array}$$

where ADI is the average daily intake (mg/kg/day^−^); *C* is the concentration of the heavy metals in soil (mg/kg); IR_ing_ is the ingestion rate (mg/day), for adults (100 mg/kg) and children (200 mg/kg); EF is the exposure frequency, 350 days/year; ED is the exposure duration, for adults (30 years) and children (6 years); SA is the exposed dermal area, for adults (5700 cm^2^) and children (2800 cm^2^); SAF is the dermal adherence factor for adults (0.07 mg cm^−2^) and children (0.2 mg cm^−2^); ABS is the dermal absorption factor, 0.001 for adults and children; IR_inh_ is inhalation rate, for adults (15 m^3^/day) and children (5 m^3^/day); PEF is the particle emission factor (1.36 × 10^9^ m^3^/kg); BW is the body weight, for adults (70 kg) and children (20 kg); and AT is the averaging time (day): for noncarcinogens, ED × 365 days, and for carcinogens (Ni and Cr) and 70 (lifetime) × 365 days [40, 41].

#### 2.6.2. Noncarcinogenic Risk Assessment

Noncarcinogenic hazards are typically characterized by the hazard quotient (HQ). The health risk from soil contamination was assessed concerning its chronic as well as carcinogenic effects, based on the calculation of ADIoral, ADIdermal, and ADIinh, plus a reference dose (RfD) that defined the toxicity values for each heavy metal, as shown in Table 1. The noncarcinogenic risk was calculated by the hazard quotient (HQ) as follows:

(9) $$\begin{array}{c} \text{HQ}=\frac{\text{ADI}}{\text{RfD}}. \end{array}$$

Then, the individual HQ of each metal was combined for the risk assessment of the hazard index (HI).

(10) $$\begin{array}{c} \text{HI}=\sum \text{HQ}. \end{array}$$

If the HQ or HI > 1, there may be potential noncarcinogenic effects on health, while HQ or HI ≤ 1 means there is no experience of any health risks for exposure by noncarcinogenic metals [42, 43].

#### 2.6.3. Carcinogenic Risk

Carcinogenic risk (CR_*i*_) was estimated as the incremental probability of developing cancer during a lifetime due to exposure to a potential carcinogen (USEPA [43]) as follows:

(11) $$\begin{array}{c} {\text{CR}}_{i}={\text{ADI}}_{i}\times {\text{SF}}_{i}, \end{array}$$

where SF_*i*_ is the cancer slope factor (mg/kg/day) through oral ingestion, dermal contact, and inhalation of each metal, as illustrated in Table 1.

(12) $$\begin{array}{c} \text{LCR}=\sum {\text{CR}}_{i}. \end{array}$$

If the value of CR and LCR exceeds 1 × 10^−4^, it represents a lifetime carcinogenic risk to the human body [44, 45].

## 3. Results and Discussion

### 3.1. Concentrations of Heavy Metal

The concentrations of Pb, Ni, Cd, Mn, Fe, Cr, and Al in groundwater exceeded both WHO and USEPA standard limits, as shown in Table 1. Besides, the levels of Zn, Pb, Ni, Cd, Mn, Fe, Cr, Al, and Cu in groundwater increased sharply from May to October, as presented in Figure S1. The heavy metal concentration in groundwater is significantly affected by leachate percolation and particularly inadequate leachate management from nonengineered municipal solid waste dump [46, 47]. In Thailand, there are three seasons, including summer (February to May), rainy (May to October), and winter (October to February) [48]. Han et al. reported that rainfall in the wet season expedites the leaching pollution from polluted topsoil. Furthermore, it can increase the outflow and permeability of leachate to some extent as rainfall increases [49]. Besides leachate percolation being dependent upon the rainfall, several factors such as the chemical constituent of the leachate, length, and deepness of the pond from the landfill also affect the extent of the contamination in groundwater [50]. Consequently, all the determined heavy metal concentrations in the groundwater are elevated in the wet season.

In soil, the average Cd (5.2 ± 1.6 mg/kg) and Cu (171.2 ± 329.4 mg/kg) concentration exceeded the standard levels of natural soil (Cd = 0.35 mg/kg and Cu = 30 mg/kg) by 15 and 6 times, respectively, as presented in Table 1. The levels of Cd and Cu may be related to the high levels of these metals in the waste which are disposed of at the landfill [51]. Mekonnen et al. found that Pb, Cd, Mn, Ni, Cu, and Zn were found in the leachate water [52]. Moreover, approximately 62% of the total fresh municipal solid waste was found to be combustible materials including plastics, paper, yard waste, textile, cardboard, rubber, and coconut husks [51]. However, Long et al. found that the kitchen waste, cinder, plastic, and paper have high globally in municipal solid waste, which accounted for 55.1–95.5% and also the main sources of Cu (41.2–1643.7 mg/kg) and Zn (109.3–1077.9 mg/kg) in the gross municipal solid waste sample [53]. The most likely sources of Cd in municipal solid waste are plastics and pigments, in which solid waste at an unlined landfill included soft plastics for 33% and hard plastics for 18.6% [47, 54]. Therefore, this metal in municipal solid waste may initiate the potential risk of soil and groundwater pollution.

The concentration of the metals during each month was a different pattern, as shown in Figure S2. However, the binding strength of Cu, Mn, and Zn in the soil of the dumping site had a uniform trend [55]. Prechthai et al. reported that Zn was found to be the highest constituent metal when compared to Mn, Cu, Cr, Cd, Pb, and Ni in a solid waste dumpsite in Nonthaburi Province, Thailand [56]. Mn, Zn, and Cd were usually found in an attenuated form that is easily leached, while Cu and Cr were found prominently in the oxidized form and stable under anaerobic conditions; Pb and Ni were present in the residual inert form [56]. The total metal content in the soil in descending content order was Fe, Al, Cu, Mn, Zn, Cr, Pb, Ni, and Cd; this was similar to the topsoil layer (0–15 cm) in 15 urban solid waste landfills (over 20 years old) in Spain [57]. The vertical permeation of the leachate and the hydrography groundwater regimen has greatly affected contaminants spreading in the soil underneath the landfill [57]. However, the age of the waste and landfill, properties of waste, local conditions, and dilution procedure have a critical impact on regional leachate quality [58, 59].

### 3.2. Water Quality

Interestingly, the WQI in groundwater around this open dump was 1038.7, which is unsuitable for consumption (WQI > 100), as shown in Figure S3. The SI of this study was Cd > Pb > Cr > Al > Ni > Mn > Fe, and Cu > Zn, respectively. Teta and Hikwa reported that groundwater from nearby boreholes within a range of 0.8–2.1 km had high levels of Pb and Cd which had a negative relationship to away from the landfill (*p* < 0.01) [47], indicating contamination from the landfill. Generally, groundwater contamination most appears within 1 km of the landfill, and the most serious in groundwater is that further contamination appears at the initial landfill stage and continues to occur for 5 to 20 years, peaking some years afterward [49]. In this study, the groundwater contamination was found within 2 km, and the landfill was >15 years old. Therefore, the groundwater around this landfill was unsuitable for consumption. The natural geology, hydrogeological conditions, the characteristics of leachate, a natural-gradient dispersion, the localized sources of pollution such as agricultural activities, municipal origin waste with batteries, paint products, and metal items cause natural attenuation to occur in the soil that may then affect the elements in aquifers, including dilution, sorption, ion exchange, precipitation, degradation processes, and redox reactions that may influence groundwater quality [1, 60].

### 3.3. Heavy Metal Pollution Levels in the Soil

**Figure 2:**
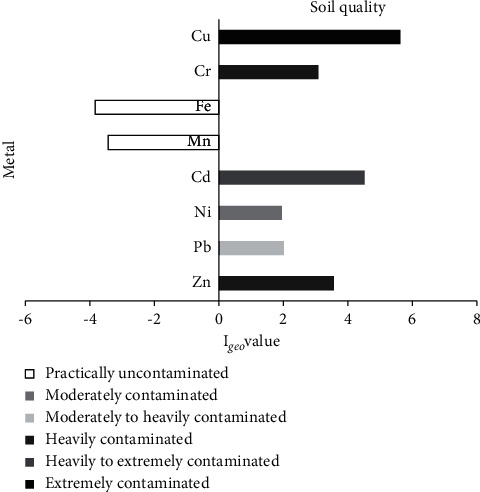
The *I*_*geo*_ value of Zn, Pb, Ni, Cd, Mn, Fe, Cr, and Cu in the soil collected in the open dump area.

The *I*_*geo*_ of Zn (3.57) and Cr (3.09) were heavy pollutions, the Pb (2.02) and Ni (1.95) were moderate, Cd (4.52) was heavy to extremely, and Cu (5.63) was extremely contaminated, as shown in Figure 2. Thus, the metal pollution levels in soil in descending order were Cu > Cd > Zn=Cr > Pb > Ni. The behavior of heavy metal in soil depends on the nature and quantity of heavy metal and is also vastly diverse across countries [61, 62]. Heavy metal can be transferred from the soil and groundwater into plants, microbes, invertebrates, animals, and humans [63]. Zinc exerts an adverse effect on the physicochemical properties of soil and plant development, including seed germination, photosynthesis, and growth rates [64, 65]. Zn can also influence the resistance of organotrophic bacteria, actinomyces and fungi, microbial respiration rates, microbial metabolic activity, and genotoxic effect [66, 67]. In vitro, Ni affects spermatozoa motility and spermatozoa membrane integrity of bovine [68]. Pb is toxic to invertebrates such as aphids and ladybirds. Earthworms are particularly sensitive to Pb [69, 70].

In both soil and water, Cd is relatively more mobile than other heavy metals (Cd > Ni > Zn > Mn > Cu > Pb = Cr) [71]. Additionally, many studies suggest that the concentration of Cd in vegetables and plants, such as rice, is related to the concentration of Cd contamination in the soil, which affects the renal function in humans [72, 73]. Additionally, Cd may be accumulated in the chloroplasts and impact the chloroplast processes in barley and maize [74]. Organisms in soil, earthworms, isopods, and gastropods are at risk of Cd accumulation and biomagnification in the soil food web [75].

However, excess Fe, Mn, Zn, and Cu may affect resistance to *Legionella pneumophila* and *Mycobacterium tuberculosis* [76, 77]. In plants, Cr (VI) at 2.5 mg/L influences the performance and structure of bacteria in aerobiotic activated sludge reactors and is associated with oxidative stress in legume plants and rhizobium symbiosis [78, 79]. In this study, the concentration of Cr was 43.30 ± 4.8 mg/kg, which may be dangerous to plants and bacteria in the soil around the landfill.

Ibrahim et al. reported that Cd and Cu exposure at 2–4 mg/L of Cd and 70–140 mg/L of Cu affected plant growth [80]. Interestingly, Cd (5.2 ± 1.6 mg/kg) and Cu (171.2 ± 329.4 mg/kg) levels in this landfill would be toxic to plants. Furthermore, Cu, Ni, and Cr can be considered as having chronic toxicity and ecotoxicity for terrestrial invertebrates, particularly in springtail [81, 82]. Moreover, Miranda et al. reported that the accumulation of Cu and Ni concentrations in the liver, kidney, and muscle of cattle was significant from the Cu and Ni in the soil and forage [83]. Zn, Pb, Ni, Cd, Mn, Fe, Cr, Al, and Cu can become residue in the blood and raw bovine milk due to pasturage and silage related to water and elements in soil [84, 85]. Zhou et al. found positive correlations for Cd and Cr between milk and silage, as well as between milk and soil [86]. Therefore, plants and organisms such as earthworms, bacterial rodents, and particularly animal livestock such as cattle may be harmed by ingesting groundwater while grazing on grass associated with soil in the vicinity of this landfill.

### 3.4. The Potential Ecological Risk Assessment

**Figure 3:**
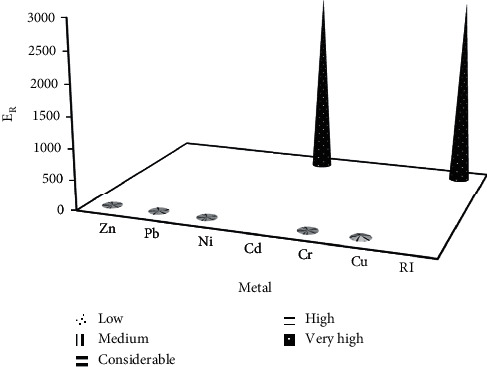
The pollution degree of Zn, Pb, Ni, Cd, Cr, and Cu and potential ecological (RI) values.

The order of the potential ecological risk factor (E_R_) of heavy metals in soil was Cd > Cu > Pb > Ni > Cr > Zn, as shown in Figure 3. Additionally, the calculated pollution degree of Cd (2,898.9) fell in the very high range, and the calculated RI was 2,945.6, which indicated an extremely high-risk degree, as shown in Figure 3. Vongdala et al., 2018, reported that Cd and Cu cause the high ecotoxicological risk level in landfill soils and surrounding areas [87]. Moreover, Ihedioha et al. found that Cd contributed 98–99% of the total potentially ecological risk in a municipal solid waste open dump in Nigeria [88]. Meanwhile, the E_R_ of Cd also showed the highest at 540 in E-waste dumpsites in Nigeria [89]. Therefore, Cd should be received overwhelming attention as an ecological hazard and be regarded as a priority pollutant in this landfill.

### 3.5. Noncarcinogenic Risk Assessment

**Figure 4:**
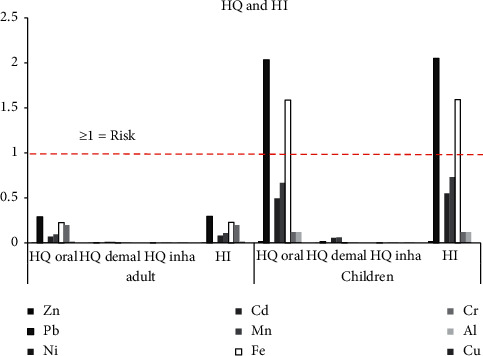
Hazard quotient (HQ) and hazard index (HI) of Zn, Pb, Ni, Cd, Mn, Fe, Cr, Al, and Cu for oral, dermal, and inhalation of the contaminated soil samples.

The HQ_oral_, HQ_dermal_, HQ_inha_, and HI of Zn, Pb, Cd, Mn, Fe, Al, and Cu in the soil were greater in children than in adults, whereas the HQ_oral_, HQ_dermal_, HQ_inha_, and HI of Ni and Cr in adults were greater than in children, as shown in Figure 4. Furthermore, the HI in children was in descending order Pb > Fe > Mn > Cd > Cu > Al > Cr > Zn > Ni, while the HI in adults was Pb > Fe > Cr > Mn > Cd > Cu > Al > Ni > Zn, respectively. Surprisingly, the HI of Pb (2.05) and Fe (1.59) in children was higher than 1, which may have a potential noncarcinogenic effect on children's health. Additionally, the risk level from Pb and Fe was level 3 (medium risk). Therefore, both the chronic risk and cancer risk by Pb and Fe for children was medium, and the calculated cases of cancer occurrence by Pb and Fe for children in this study were >1 per 100,000 inhabitants but <1 per 10,000 inhabitants, respectively [42]. Incidentally, children living nearby a municipal waste incinerator also suffer from the burdens of Pb, Cd, and Cr associated with genotoxicity and epigenetic modifications [90]. Both chronic toxicity and acute toxicity by Pb exposure have the potential of deleterious systemic effects including immune imbalances, hypertension, frank anemia, intellectual disability, gastrointestinal effects, skeletal delay, development of deciduous dentistry, vitamin *D* deficiency, surrogate for Ca, infertility, and hearing loss [91, 92]. Kim and Williams reported that landfills are high-risk areas for environmental Pb exposure for children living in poor areas in many countries [93]. Blood Pb levels of children living near landfills were related to the increase of Pb levels in the soil. Also, Dórea reported that Pb also causes increased neurological health issues from prenatal exposure to portend and negative results were commonly in infants (<6 months) [94]. In children, Fe overload causes *β*-Thalassemia in anemic patients [95]. Thus, Pb and Fe contamination in the soil of this landfill has the potential effect of being a chronic risk, especially to children's health.

### 3.6. Carcinogenic Risk Assessment

**Figure 5:**
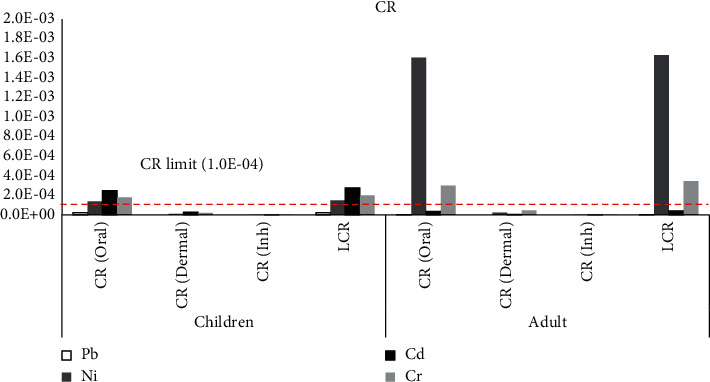
Carcinogenic risks (CRs) of Pb, Ni, Cd, and Cr for oral, dermal, and inhalation of the contaminated soil samples.

The CR_oral_ and LCR of Pb and Cd in children were greater than adults, whereas the CR_oral_ and LCR of Ni and Cr in adults were greater than children. Further, the *CRd*_ermal_ and *CRd*_Inh_ of Ni and Cr in adults were also higher than children, while both *CRd*_ermal_ and CR_dInh_ of Cd in children were higher than adults, as shown in Figure 5. Surprisingly, the CR_oral_ of Ni, Cd, and Cr in children was 1.4*E* − 04, 2.5*E* − 04, and 1.8*E* − 04, respectively, and the LCR of Ni, Cd, and Cr in children was 1.5*E* − 04, 2.8*E* − 04, and 2.0*E* − 04, respectively, in adults. The CR_oral_ of Ni and Cr was 1.6*E* − 03 and 3.0*E* − 04, respectively, while the LCR of Ni and Cr was 1.6*E* − 03 and 3.4*E* − 04, respectively. Both exceeded the carcinogenic risk limit at 1.0*E* − 04 and indicated that a lifetime carcinogenic posed a risk to both children and adults. Nickel is a major carcinogen to humans and up-take is through the respiratory tract, digestive system, and skin [96]. The primary target organs are the kidneys and lungs [97]. Exposure to Ni is intricately linked to an increased risk of human lung and nasal cancer [98, 99]. Furthermore, Ni is the most common cause of contact allergies and causes dermatitis worldwide, commonly found in children [100, 101]. Jacob et al. confirmed children older than six months may have suspected Ni allergic contact dermatitis [102]. Moreover, congenital heart defect occurrence in offspring may be associated with Ni exposure in mothers [103]. This study indicates that it may be harmful to adult workers, their children, and any other people living with Ni in the area.

Hexavalent chromium (Cr^+6^) compounds are classified as carcinogenic to humans in the respiratory tract, giving rise to cancer of the lungs, nose, and nasal sinuses. It is mutagenic when inhaled and also potentially when ingested orally in large quantities [104, 105]. In particular, in certain occupational environments, workers may suffer from inhalation exposure by Cr^+6^ dust, mists, and fumes [106]. Environmental exposure to Cr^+6^ negatively affects the outcome of the pregnancy and consequently the health of two generations, resulting in higher pregnancy loss, spontaneous miscarriage, and low birth rate [107]. Besides, Cr may affect fetal growth; particularly, early and midterm pregnancies appear to be the riskiest periods for fetal exposure to Cr [108]. Children of Cr^+6^ exposed women experience increased respiratory problems, perinatal jaundice, and congenital disabilities [107].

Cadmium has been defined as being carcinogenic to both animals and humans [109, 110]. Both Cd exposure through occupation and environment leads to tumorigenesis and increases the risk of various cancers, particularly lung, prostate, jugular, and pancreatic cancer [111–113]. They also reported that long-term Cd exposure could promote breast cancer. Early life low levels of Cd exposure are related to lower child IQ in 5-year-old girls and boys [114], and 10-year-old children with Cd exposure are associated with lower ingeniousness, especially in boys [115]. This study indicated that children might suffer a detrimental health effect due to Cd and Cr exposure. However, the limitation of this study was to find out the worker health profiles and the difficulty of the database of air pollution reports and local waste management policy and plans. Therefore, intensive environmental health risk surveillance needs long-term operations.

## 4. Conclusions

In this study, the average concentrations of Pb, Ni, Cd, Mn, Fe, Cr, and Al in groundwater exceeded both WHO and USEPA standards. Besides, the levels of Zn, Pb, Ni, Cd, Mn, Fe, Cr, Al, and Cu in groundwater elevated sharply from April to October, indicating these metals levels increased mostly in the wet season. The WQI in the groundwater around this landfill is unsuitable for consumption and is due to groundwater contamination, which mainly appears within 1 km of the landfill and is especially impacted by the landfill's age. The average Cd and Cu concentrations exceeded the average in natural soil. The *I*_*geo*_ of Cd was heavy to extremely contaminated, and lastly, the Cu was extremely contaminated. Ultimately, the metal transfer from the soil and groundwater may pose serious health problems to humans and livestock, especially cattle. The degree of pollution for Cd was very high and the RI indicated that the risk degree was very high. Thus, Cd should be provoking a great deal of attention as an ecological hazard and be considered a priority pollutant in this area.

The HQ and HI of Zn, Pb, Cd, Mn, Fe, Al, and Cu in the soil for children were greater than adults; the HQ and HI of Ni and Cr in adults were greater than in children. The HI of Pb and Fe in children was higher than 1, which was interpreted as a noncarcinogenic effect on children and indicated that both metals (Pb and Fe) could cause medium levels of chronic risk and cancer risk in children. The CR_oral_ and LCR of Pb and Cd in children were greater than adults, whereas the CR_oral_ and LCR of Ni and Cr in adults were greater than children. The CR_oral_ and LCR of Ni, Cd, and Cr in children and the CR_oral_ and LCR of Ni and Cr in adults exceeded the carcinogenic risk limit. Thus, the data indicated a lifetime carcinogenic risk to both children and adults. The environmental surveillance of heavy metals as pollutants must be addressed and monitored to mitigate the health risks, such as through the application of soil amendments and risk control measures, continuous monitoring of the groundwater, soil and leachate treatment processes, operating blood and urine screening of workers at the landfill, and exposure avoidance for children living near the contaminated landfill area.

## Figures and Tables

**Table 1:** The average concentration, minimum, maximum, the communality (*h*^2^), relative weight (*W*), background value (*Bn*), background value (*C*_*R*_), toxic response coefficients (*T*_*R*_), reference dose (RfD), and cancer slope factor (SF) for oral, dermal, and inhalation in the groundwater and soil of Zn, Pb, Ni, Cd, Mn, Fe, Cr, Al, and Cu.

Sample		Zn	Pb	Ni	Cd	Mn	Fe	Cr	Al	Cu
Water	Mean ± SD	0.26 ± 0.02	0.18 ± 0.01	0.17 ± 0.01	0.15 ± 0.01	0.39 ± 0.10	6.85 ± 16.40	0.22 ± 0.02	0.81 ± 0.05	0.42 ± 0.11
	Min–max	0.02–0.42	ND-0.31	ND-0.30	ND-0.29	ND-2.16	0.02–58.69	ND-0.52	0.01–1.73	ND-0.73
	WHO (2011)	3	0.01	0.07	0.003	0.05	0.3	0.05	0.2 (WHO, 2010)	2
	USEPA (2012)	5	0.015	—	0.005	0.05	0.3	0.1	—	1.3
	*h* ^2^	0.95	0.88	0.86	0.76	0.21	0.07	0.84	0.86	0.97
	*W*	0.15	0.14	0.14	0.12	0.03	0.01	0.13	0.13	0.15

Soil	Mean ± SD	64 ± 38.6	29.7 ± 39.9	9.9 ± 0.99	5.2 ± 1.6	139 ± 13.5	4966.4 ± 450.8	43.3 ± 4.8	1237.1 ± 115.10	171.2 ± 329.4
	Min–max	21.3–126.03	6.9–69.70	3.7–21.9	ND-16.5	76.8–224.5	3640.7–6197.5	21.7–81.1	49.8–2715.9	10.9–685.4
	Average in natural soil^*∗*^	90	35	50	0.35	1,000	40,000	70	—	30
	*Bn* ^*∗∗*^	3.6	4.9	1.7	0.15	1,000	47,200	3.40	—	2.30
	*C*_*R*_^*∗∗∗*^	97.2	25	28.2	0.0534	—	—	66.7	—	23.1
	*T*_*R*_^*∗∗∗∗*^	1	5	5	30	—	—	2	—	5

RfD^#^	Oral	3.00*E* − 01	1.40*E* − 03	2.00*E* − 02	1.00*E* − 03	2.00*E* − 02	3.00*E* − 01	3.00*E* − 03	1.00*E* + 00	4.00*E* − 02
	Dermal	6.00*E* − 02	5.24*E* − 04	5.40*E* − 03	2.50*E* − 05	6.00*E* − 04	3.00*E* − 01	3.00*E* − 03	2.70*E* − 01	1.20*E* − 02
	Inhalation	3.00*E* − 01	3.52*E* − 03	2.06*E* − 02	1.00*E* − 03	1.43*E* − 05	NA	2.86*E* − 05	1.00*E* − 03	4.00*E* − 02

SF^#^	Oral	NA	8.50*E* − 03	1.70*E* + 00	5.00*E* − 01	NA	NA	5.01*E* − 01	NA	NA
	Dermal	NA	4.25*E* + 01	2.00*E* + 01	NA	NA	2.00*E* + 01	NA	NA	NA
	Inhalation	NA	NA	8.40*E* + 01	6.3*E* + 00	NA	NA	4.20*E* + 01	NA	NA

ND: not detected; —: not available. ^*∗*^Bowen [25], ^*∗∗*^Zarcinas et al. [26] and Klinsawathom et al. [27], ^*∗∗∗*^Hakanson [28], ^*∗∗∗∗*^Xu et al. [29] and Xv et al. [30], and ^#^USEPA [31] and USEPA IRIS [32].

## Data Availability

The data used to support the findings of this study are included within the article.

## Supplementary Materials

Supplementary Materials Supplementary Figure S1: the levels of Zn, Pb, Ni, Cd, Mn, Fe, Cr, Al, and Cu determined in groundwater compared with the WHO and USEPA standard limits. Supplementary Figure S2: the levels of Zn, Pb, Ni, Cd, Mn, Fe, Cr, Al, and Cu determined in the soil compared with natural soil standard limits. Supplementary Figure S3: the water quality index of Zn, Pb, Ni, Cd, Mn, Fe, Cr, Al, and Cu in the groundwater.
