# Supplementary material for: Carcinogenic Risk of Pb, Cd, Ni, and Cr and Critical Ecological Risk of Cd and Cu in Soil and Groundwater around the Municipal Solid Waste Open Dump in Central Thailand
Source: J Environ Public Health. 2022 Feb 28;2022:3062215. doi: 10.1155/2022/3062215 (PMC8901317; doi:10.1155/2022/3062215)
Supplement: Supplementary Materials — Supplementary Figure S1: the levels of Zn, Pb, Ni, Cd, Mn, Fe, Cr, Al, and Cu determined in groundwater compared with the WHO and USEPA standard limits. Supplementary Figure S2: the levels of Zn, Pb, Ni, Cd, Mn, Fe, Cr, Al, and Cu determined in the soil compared with natural soil standard limits. Supplementary Figure S3: the water quality index of Zn, Pb, Ni, Cd, Mn, Fe, Cr, Al, and Cu in the groundwater. [file 3062215.f1.pdf]

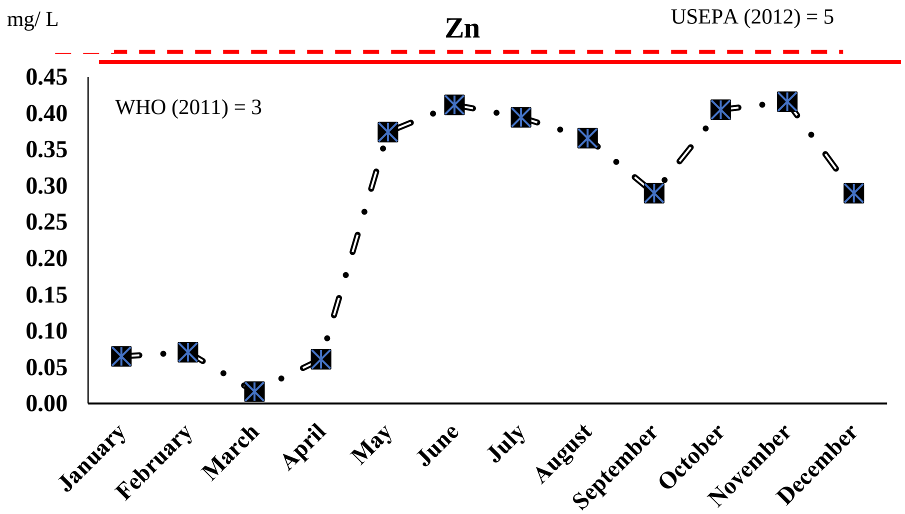

S1A

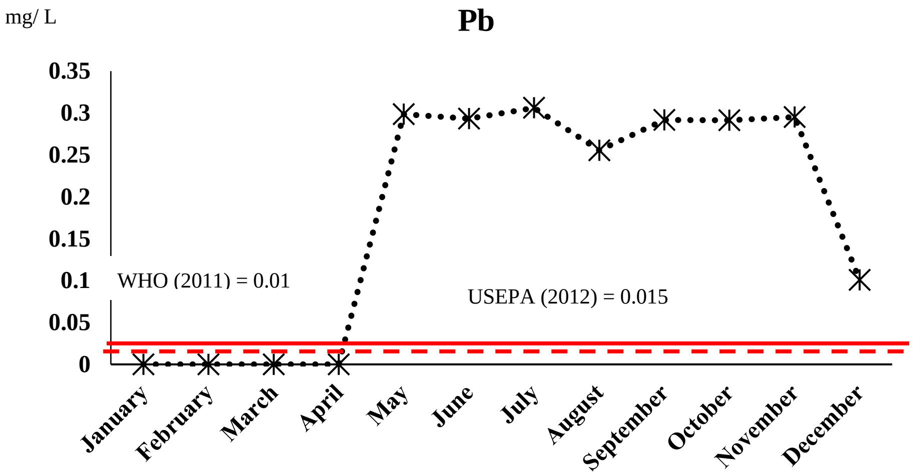

S1B

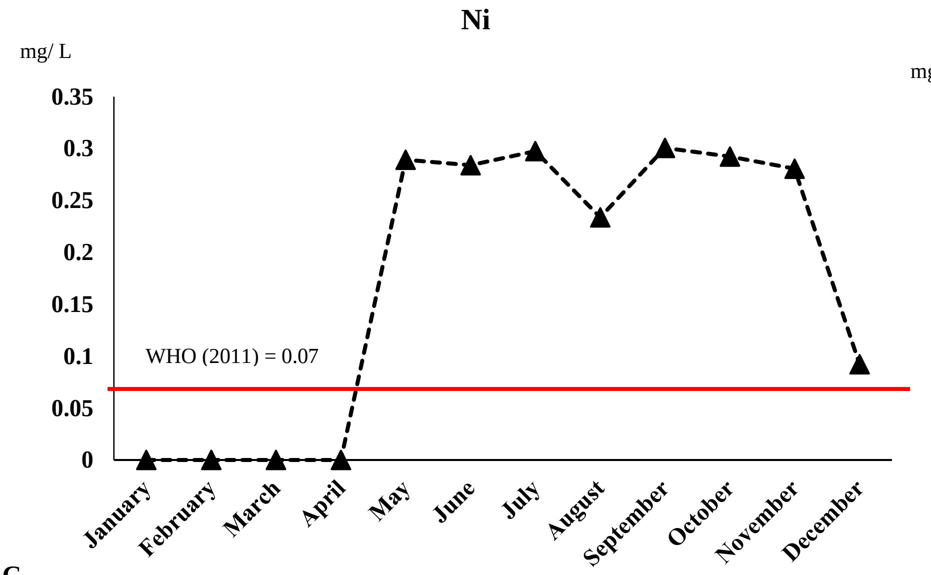

S1C

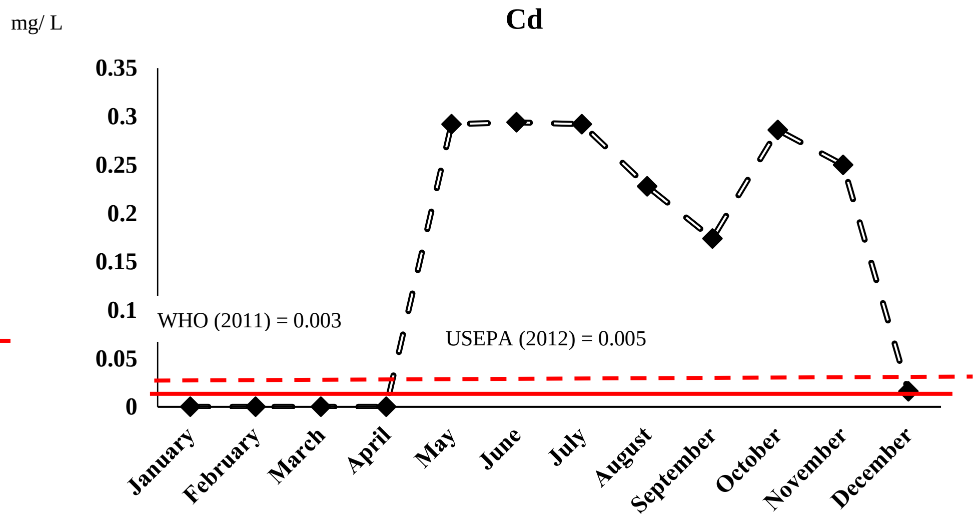

S1D

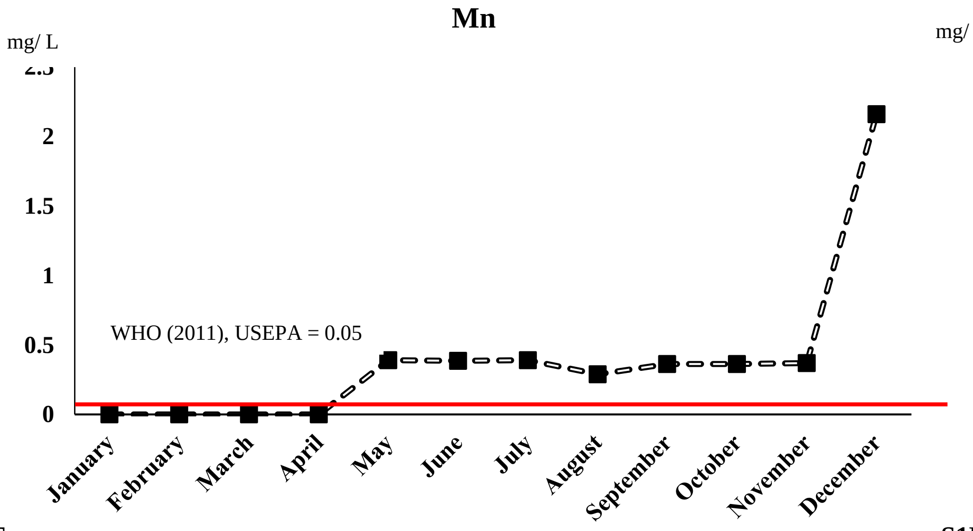

S1E

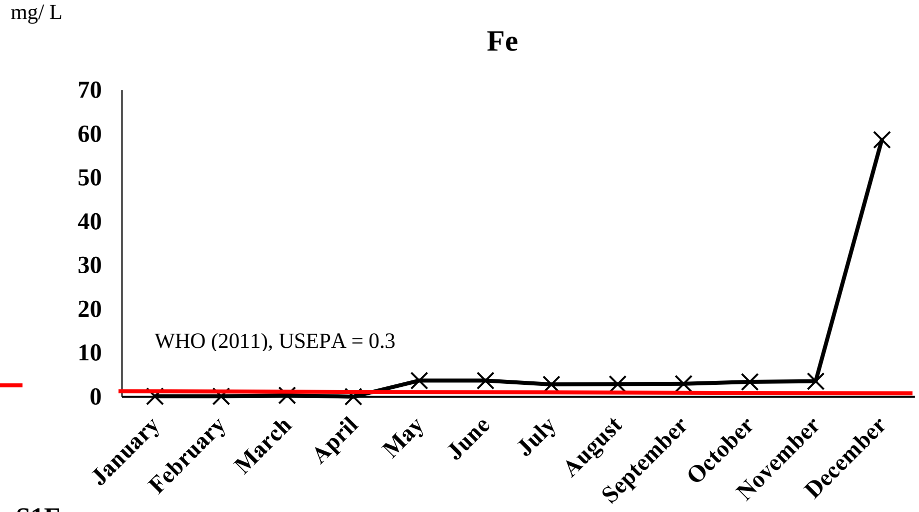

S1F

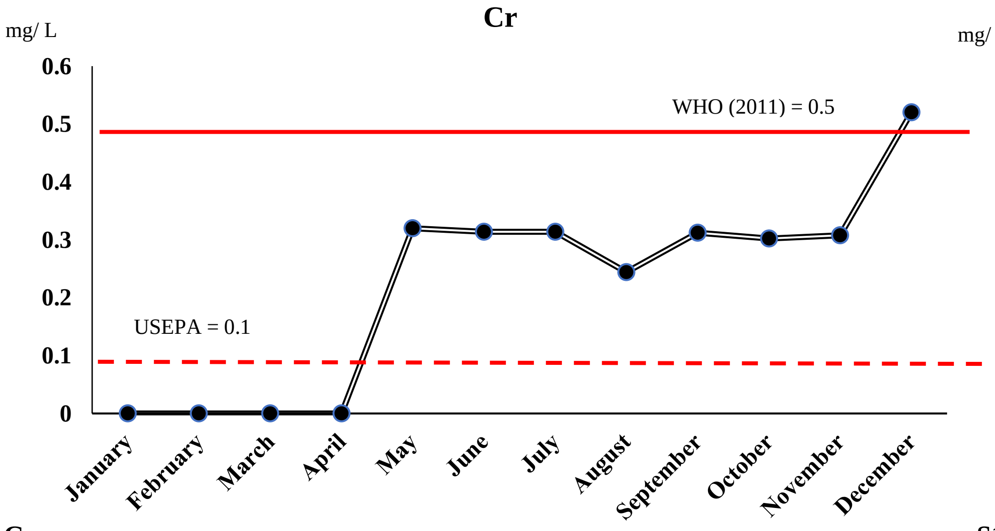

S1G

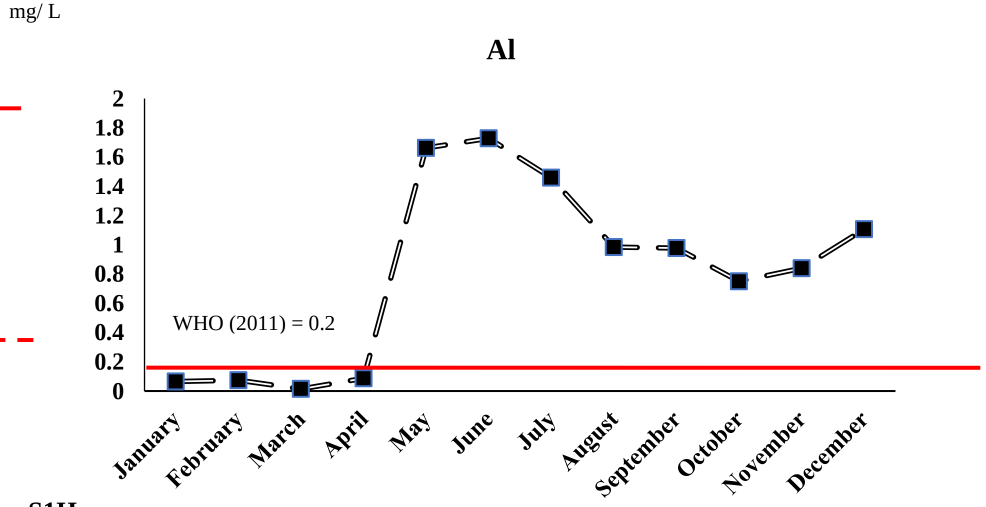

S1H

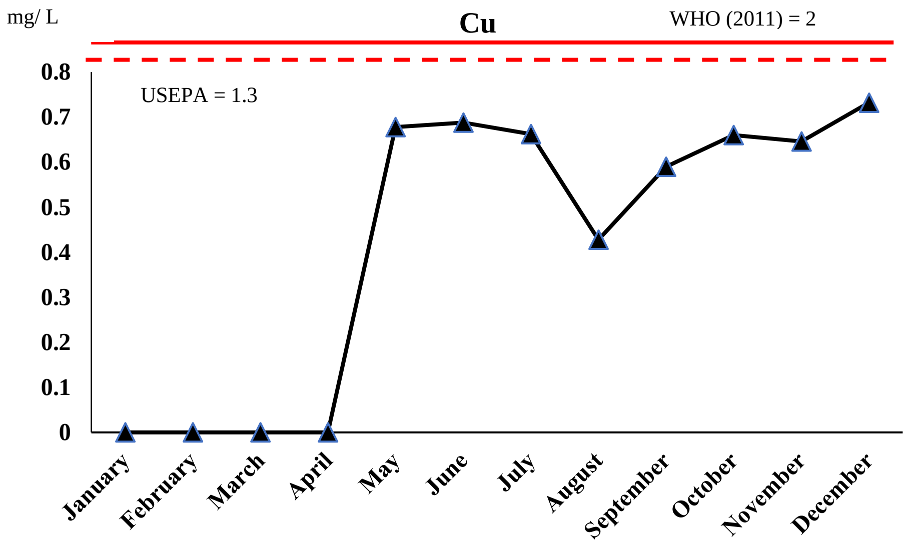

S1I

Supplementary figure S1 The levels of Zn, Pb, Ni, Cd, Mn, Fe, Cr, Al, and Cu determined in groundwater compared with the WHO and USEPA standard limits.

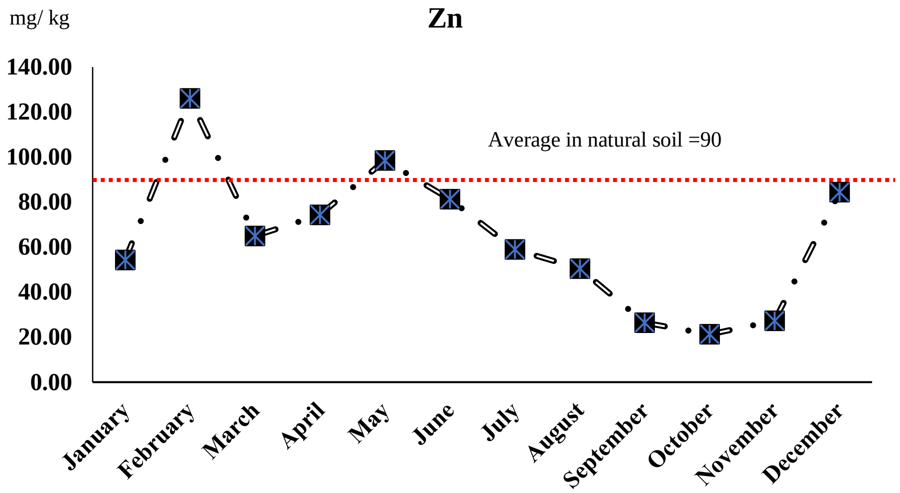

S2A

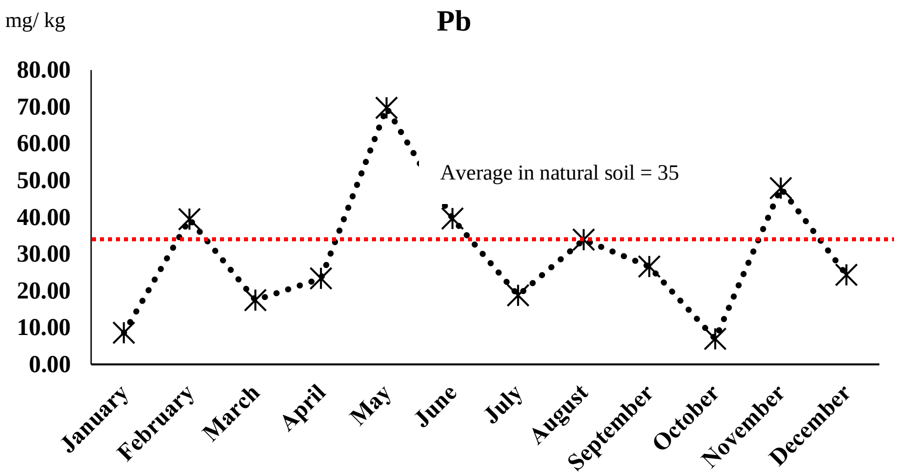

S2B

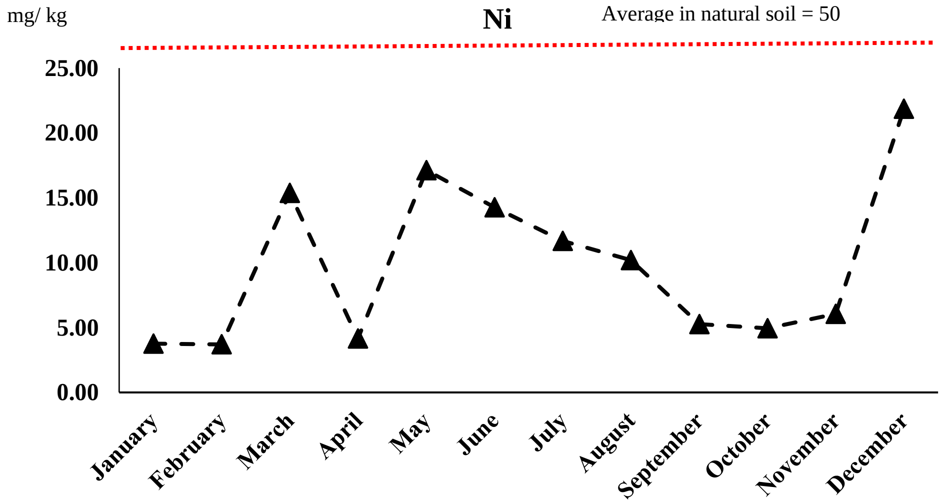

S2C

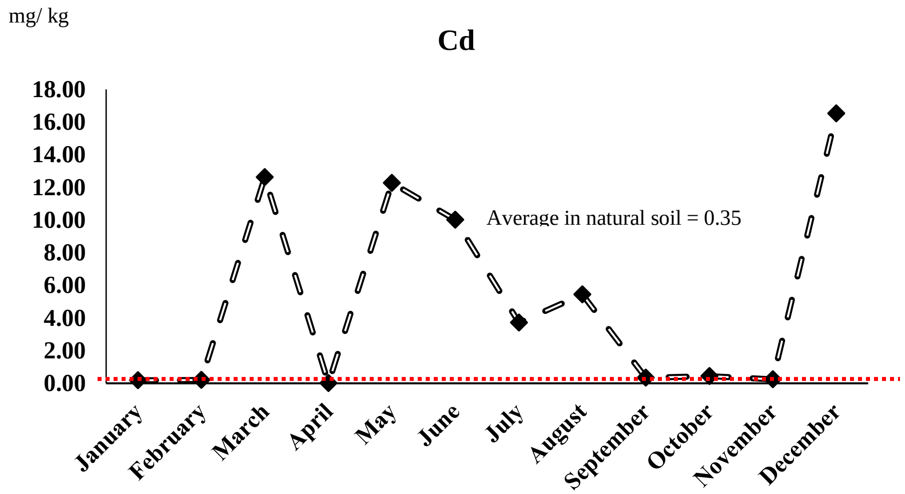

S2D

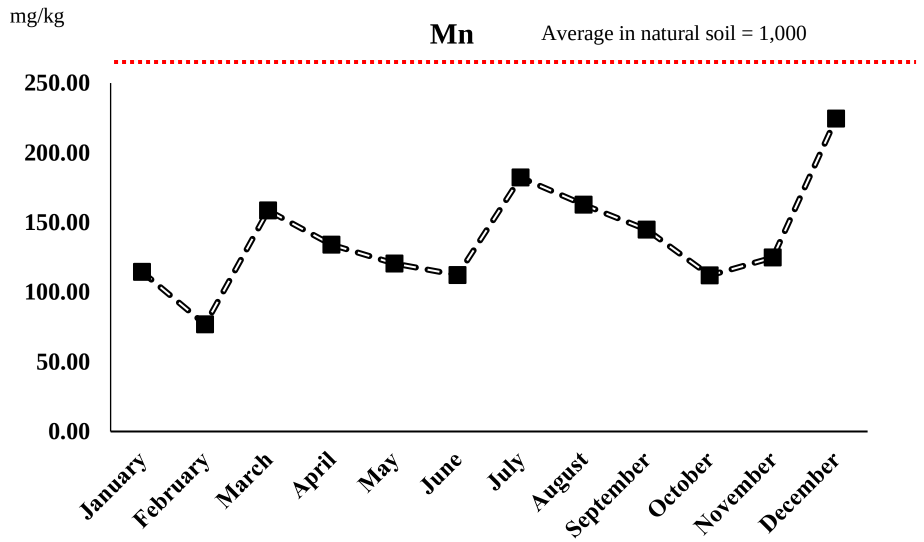

S2E

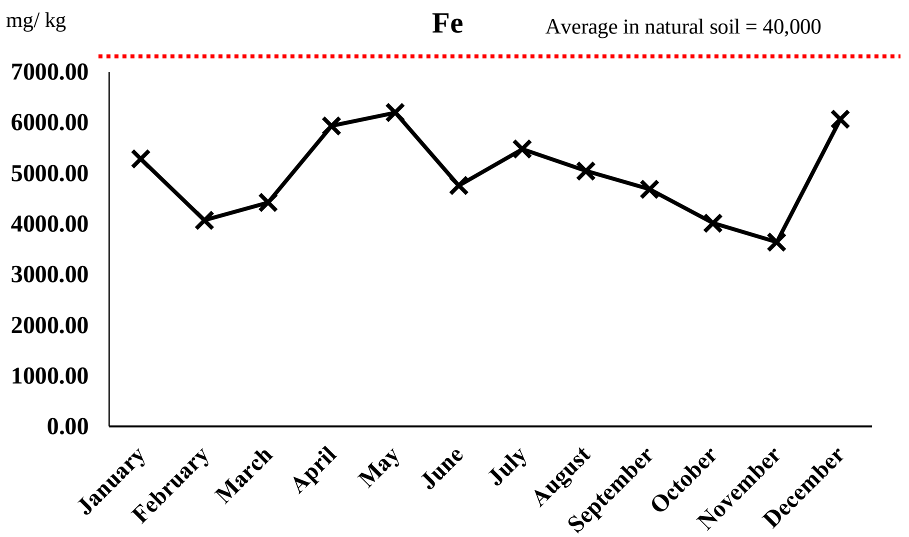

S2F

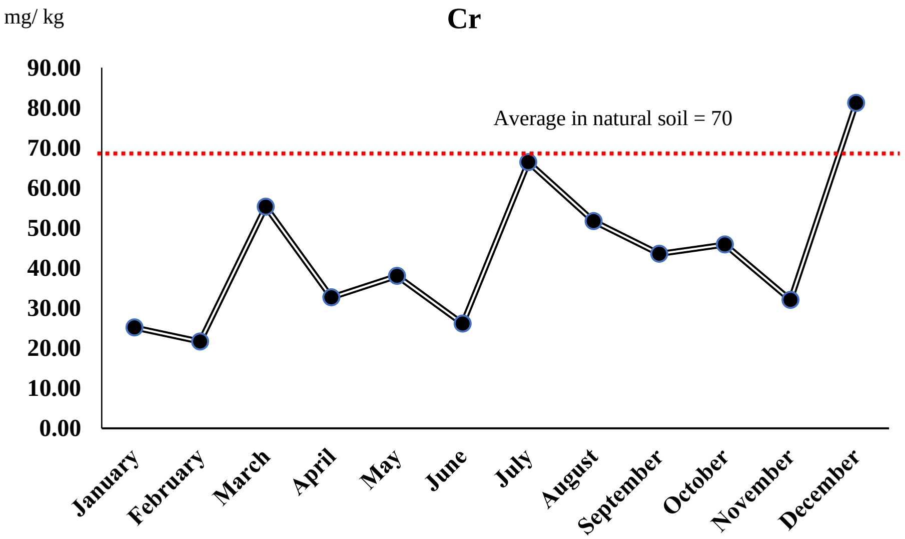

S2G

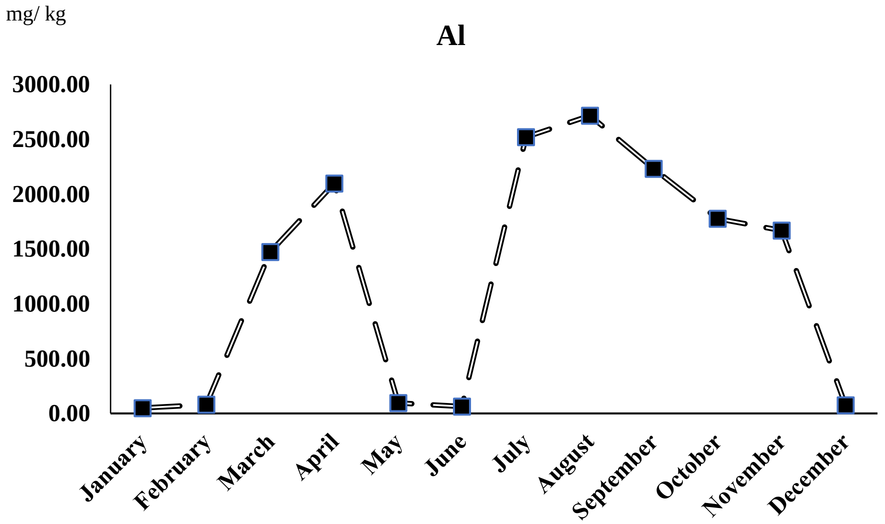

S2H

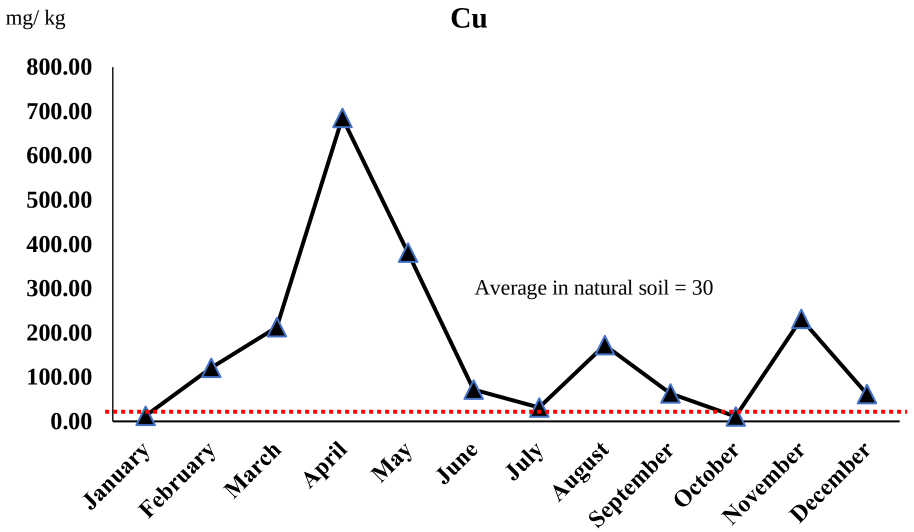

S2I

Supplementary figure S2 The levels of Zn, Pb, Ni, Cd, Mn, Fe, Cr, Al, and Cu determined in the soil compared with natural soil standard limits

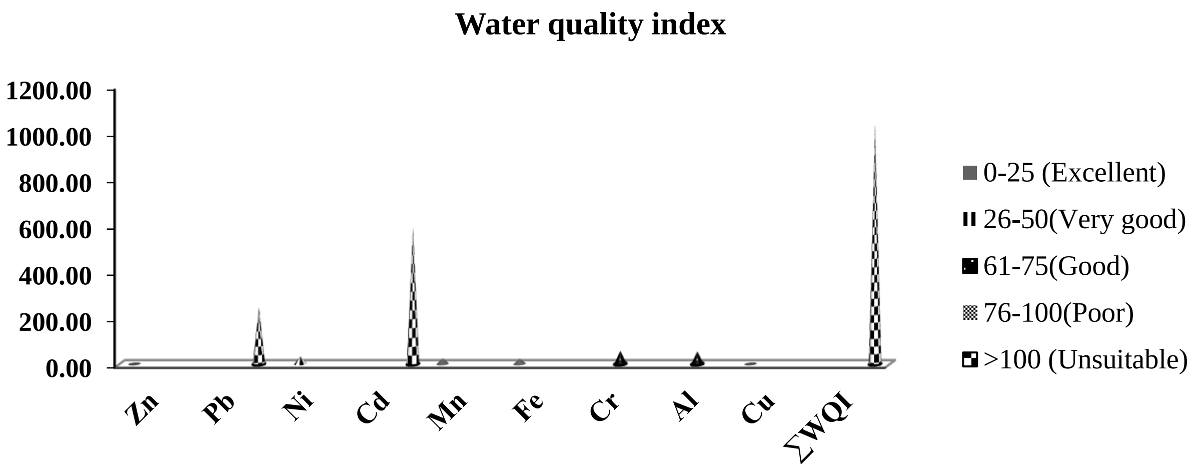

Supplementary figure 3 The water quality index of Zn, Pb, Ni, Cd, Mn, Fe, Cr, Al, and Cu in the groundwater
